# Causes and treatment strategies of unilateral leaflet escape of bileaflet mechanical prosthetic heart valves after surgery: a case series

**DOI:** 10.1186/s12872-023-03106-0

**Published:** 2023-02-07

**Authors:** Xianzhi Wang, Jiawei Qiu, Cunfu Mu, Wenlin Zhang, Chunzhu Xue, Yang He, Qinglin Mu, Chunyang Fu, Dianyuan Li

**Affiliations:** 1 Department of Thoracic and Cardiac Surgery, The First People’s Hospital of Guangyuan, Sichuan, China; 2 Fuwai Hospital, Chinese Academy of Medical Sciences, Beijing, China; 3 Department of Radiology, The First People’s Hospital of Guangyuan, Sichuan, China; 4 Department of Cardiovascular Surgery, The Affiliated Suzhou Hospital of Nanjing Medical University, Suzhou Municipal Hospital, Gusu School, Nanjing Medical University, 26 Qian Street, Suzhou, 215000 Jiangsu China

**Keywords:** Bileaflet mechanical prosthetic heart valve, Leaflet escape, Diagnosis and treatment strategy

## Abstract

**Background:**

During the eleven years from 2010 to 2021, preliminary statistics have shown that Fuwai Hospital completed 23,571 mechanical valve replacements for various types of valves, and 1139 mechanical valve replacements were performed in Guangyuan First People's Hospital. Only two patients developed valve leaflet escape, so valve leaflet escape is a rare postoperative complication.

**Case presentation:**

In 2010 and 2021, two patients were selected after they had unilateral leaflet escape after having mechanical valve replacements in Fuwai Hospital of Chinese Academy of Medical Sciences and Guangyuan First People's Hospital. Both patients underwent reoperations with the classic operation and the new bileaflet mechanical prosthetic heart valve was sutured. The treatment of detached single lobe and distal vessel was comprehensively determined, and the condition was treated according to the patient's symptoms, CT results, ultrasound results and other test results, as well as whether this detached lobe caused any abnormal hemodynamics of the distal vessel. The patient with mechanical aortic valve escape completed the 10-year follow-up, and patient with mechanical mitral valve escape completed the 3-month follow-up. there was no thrombosis or hematoma at the embolic site; the patient had no lower limb symptoms.

**Conclusions:**

The reason for the leaflet escape may be related to the valve design and the leaflet material. If the detached leaflets are damaged and if the distal blood vessels are affected, simultaneous surgical treatment is required. Those patients whose vessels were not damaged by the valve lobe should be carefully monitored.

## Background

Looking back at the history of artificial mechanical valves, there has been a development process, initially there were the first generation of cage ball valves and cage disc valves, then there were the second generation of single-leaf tilting disc valves and finally there were the third generation of bileaflet mechanical prosthetic heart valves. In 1960, the cage ball mechanical valve was the initial prosthetic that was used for heart valve replacements worldwide. In 1963, researchers in China also developed the domestic cage ball valve and applied it in the clinic. In 1969, the second generation of single-leaf tilting disc valves was developed abroad, and in 1978, the domestic single-leaf tilting disc valves were developed and eventually were widely used, with good clinical effects. Since its invention in 1980, the bileaflet mechanical prosthetic heart valve has eliminated the use of the previous two kinds of valves because the bleaflet mechanical prosthetic heart value has an excellent performance and has become the mainstream product that is used today. Barbetseas J et al. and McLintock C et al. reported that the main causes of bileaflet mechanical prosthetic heart valve replacement include postoperative valve restenosis (53.3–62.5%), prosthetic valve dysfunction (18. 6–20.4%) and prosthetic valve perivalvular leakage (5. 3–9.2%) [1–6]. During the eleven years from 2010 to 2021, preliminary statistics have shown that Fuwai Hospital completed 23,571 mechanical valve replacements for various types of valves, and 1139 mechanical valve replacements were performed in Guangyuan First People's Hospital. Only two patients developed valve leaflet escape, so valve leaflet escape is a rare postoperative complication. Nakamura et al. reported a case report of unilateral leaflet escape (Edward TEKNA, 29 mm) after bileaflet mechanical prosthetic heart valve replacement [7]. Amorós Rivera C et al. reported the survival after mitral valve replacement for leaflet escape in a contemporary On-X mechanical valve, and they introduced the clinical symptoms and treatment experience of such patients [8]. W B Hemmer et al. introduced a case of report with acute onset of severe pulmonary edema and low cardiac output. He had undergone mitral valve replacement with a TEKNA prosthesis (model 9120R, size 27 mm). Severe mitral regurgitation was diagnosed [9]. There is no any report of GKS (Beijing Star Medical Device Co., Ltd) leaflet escape at present in China. Our center, in conjunction with Fuwai Hospital, reported the diagnosis and treatment of two cases of unilateral leaflet escape and provided new ideas and experience for the diagnosis and treatment of such patients.

## Case presentation

In 2010 and 2021, two patients were selected after they had unilateral leaflet escape after having mechanical valve replacements in Fuwai Hospital of Chinese Academy of Medical Sciences and Guangyuan First People's Hospital, respectively. The patient who had aortic valve failure had surgery with GKS 23A (Beijing Star Medical Device Co., Ltd) on March 9, 2000. The patient who had mitral valve failure had surgery with GKS 25 M (Beijing Star Medical Device Co., Ltd.) on January 5, 2008. The selected patients both had sudden and severe heart failure after undergoing bileaflet mechanical prosthetic heart valve replacements for valvular heart disease, and the patients had indications for second valve replacement surgeries. There was close communication with the patients and their families before the surgeries, and the patients signed the informed consent form for the operations.

The patient who had aortic valve failure had surgery on March 9, 2000, and the replacement valve was a GKS 23A (Beijing Star Medical Device Co., Ltd). The reoperation surgery was on April 8, 2010, and the replacement valve was a Standard A 23 (CarboMedics Inc.). The patient who had mitral valve failure had surgery on January 5, 2008, and the replacement valve was a GKS 25 M (Beijing Star Medical Device Co., Ltd.). The reoperation surgery was on August 8, 2021, and the replacement valve was an F7-025 (Sorin Group Italia S.R.L.).

Both patients underwent reoperations with the classic operation. All patients underwent surgery using the original incision with a median sternotomy approach; the chest incision was made with swing saw; and the patients were prepared for femoral artery and vein catheterization. Cardiopulmonary bypass was established for both of the patients after the thoracotomies. To complete the operation, any pericardial adhesions were separated. Catheterization of the aorta and right atrium or the superior and inferior vena cava was established, and left ventricular drainage was established. After blocking the aorta and establishing cardiac arrest, the heart was explored, the damaged mechanical valve was dismantled, the valvular ring diameter was measured, the appropriate valve was selected, and the new bileaflet mechanical prosthetic heart valve was sutured. The treatment of detached single lobe and distal vessel was comprehensively determined, and the condition was treated according to the patient's symptoms, CT results, ultrasound results and other test results, as well as whether this detached lobe caused any abnormal hemodynamics of the distal vessel. Ultrasound and CT examinations were performed before the patients were discharged, and follow-up visits were conducted in the outpatient department 3 months after the operations. Then, the ultrasound and CT were reviewed.

The patient with aortic valve failure had severe aortic regurgitation, and the patient with mitral valve failure had severe mitral regurgitation (Table 1). The 2 patients occurred leaflet escape, and all the valves had thrombosis around. The right coronary sinus of aortic valve patients is obviously dilated (19*18 mm cystic echo area). The left atrium of mitral valve was obviously dilated. There was no perivalvular leakage in both patients. The perivalvular tissue of both patients proliferated obviously, and the valve annulus, the remaining valve leaflets and the valve sutures were not obviously damaged. Both patients used mechanical heart valves (GKS) (Table 2 and 3). The indications for the valve replacements were clear in both of the patients, and the surgical risk for both patients was assessed clinically as medium risk.

**Table 1:** Clinical baseline data of the 2 patients who had unilateral leaflet escape of the bileaflet mechanical prosthetic heart valves after surgery

Variable	Aortic valve failure patient	Mitral valve failure Patient
Male	Male	Female
Age (years)	32	48
Hypertension	None	None
Coronary heart disease	None	None
Apoplexy	None	None
Time from the first valve replacement to the second valve replacement (years)	10	13
STS score (%)	3.5	4.3
NYHA cardiac function classification	II	II
Aortic regurgitation flow (ml/beat)	72.2	4.3
Aortic regurgitation fraction (%)	62.1	4.2
Mitral regurgitation (ml/beat)	3.0	62.5
Mitral regurgitation fraction (%)	4.3	67.2
INR(R)	2.0	2.2
BNP(pg/ml)	5600	3046

*STS* Society of Thoracic Surgeons, *NYHA* New York Heart Association, *INR* International standard ratio of prothrombin time, *BNP* terminal pro-brain natriuretic peptide

**Table 2:** 2 Mechanical valve status of patients undergoing re-valve replacement surgery

Variable	Aortic valve failure patient	Mitral valve failure Patient
Unilateral leaflet sheddingescape	Y	Y
Valve thrombosis	Y	Y
Aortic sinus dilatation	Y	N
Perivalvular leakage	N	N
Perivalvular tissue hyperplasia	Y	Y
Residual leaflet breakage	N	N
Rupture of valve ring	N	N
Valve suture shedding	N	N
Valve brand	GKS	GKS

*GKS* Beijing Star Medical Device Co., Ltd

**Table 3:** Perioperative displaced valve leaflets of patients undergoing re-valve replacement surgery

Variable	Aortic valve failure patient	Mitral valve failure patient
Embolism site of escape leaflet	Left common iliac artery	Right common iliac artery
Embolic site velocity(cm/s)	123.0	118.0
Thrombosis adhesion	N	N
Diameter of blood vessel at embolization site (mm)	12.1	14.2
Hematoma at embolic site	N	N
Limb lameness	N	N
Affected limb ABI	1.0	1.1

*ABI* Ankle-brachial index

**Fig. 1:**
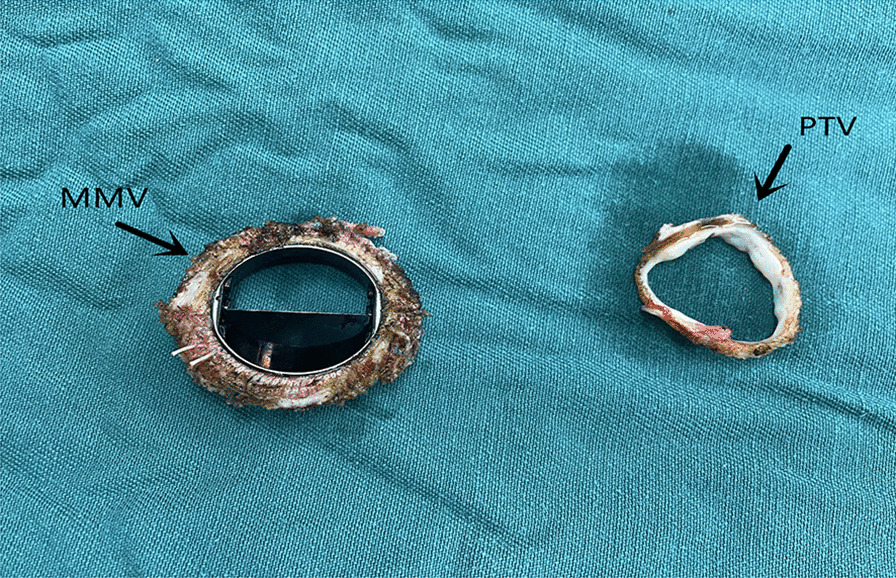
Physical drawing of a dismantled bileaflet mechanical valve. When the mechanical valve was removed, one lobe of the valve was missing, and the proliferative tissue around the valve had a complete annular structure. MMV: Mechanical mitral valve; PTV: Proliferative tissue of valve

**Fig. 2:**
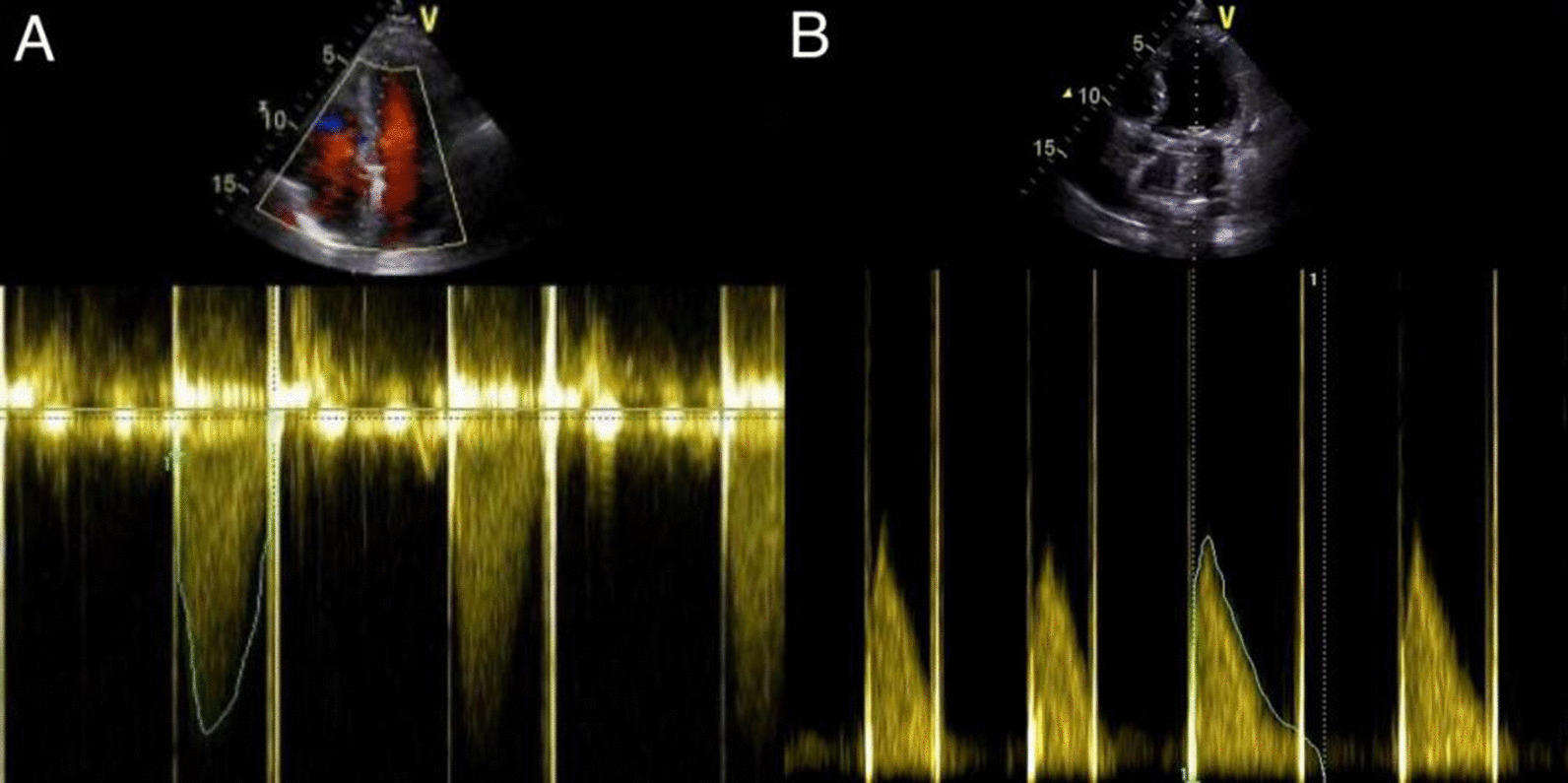
Post-operative echocardiographies of the patients with aortic valve and mitral valve replacements. **A** The echocardiography after aortic valve replacement shows that the forward blood flow is normal, with no obvious regurgitation or perivalvular leakage; **B** The echocardiography after mitral valve replacement shows that the forward blood flow is normal without obvious regurgitation or perivalvular leakage

The operations in both of the patients were successful, and there were no complications. See Fig. 1 for the techniques that were used to remove the mechanical valves. Postoperative cardiac ultrasounds showed normal blood flow in both of the patients (Fig. 2). The aortic valve failure patient's valve leaflet had migrated into the left common iliac artery, and the mitral valve failure patient's valve leaflet had migrated into the right common iliac artery. The flow velocity at the embolic site for the patient with aortic valve failure was 123 cm/s, the patient with mitral valve failure was 118 cm/s and no hematoma was formed at the embolic site in either patient.

**Fig. 3:**
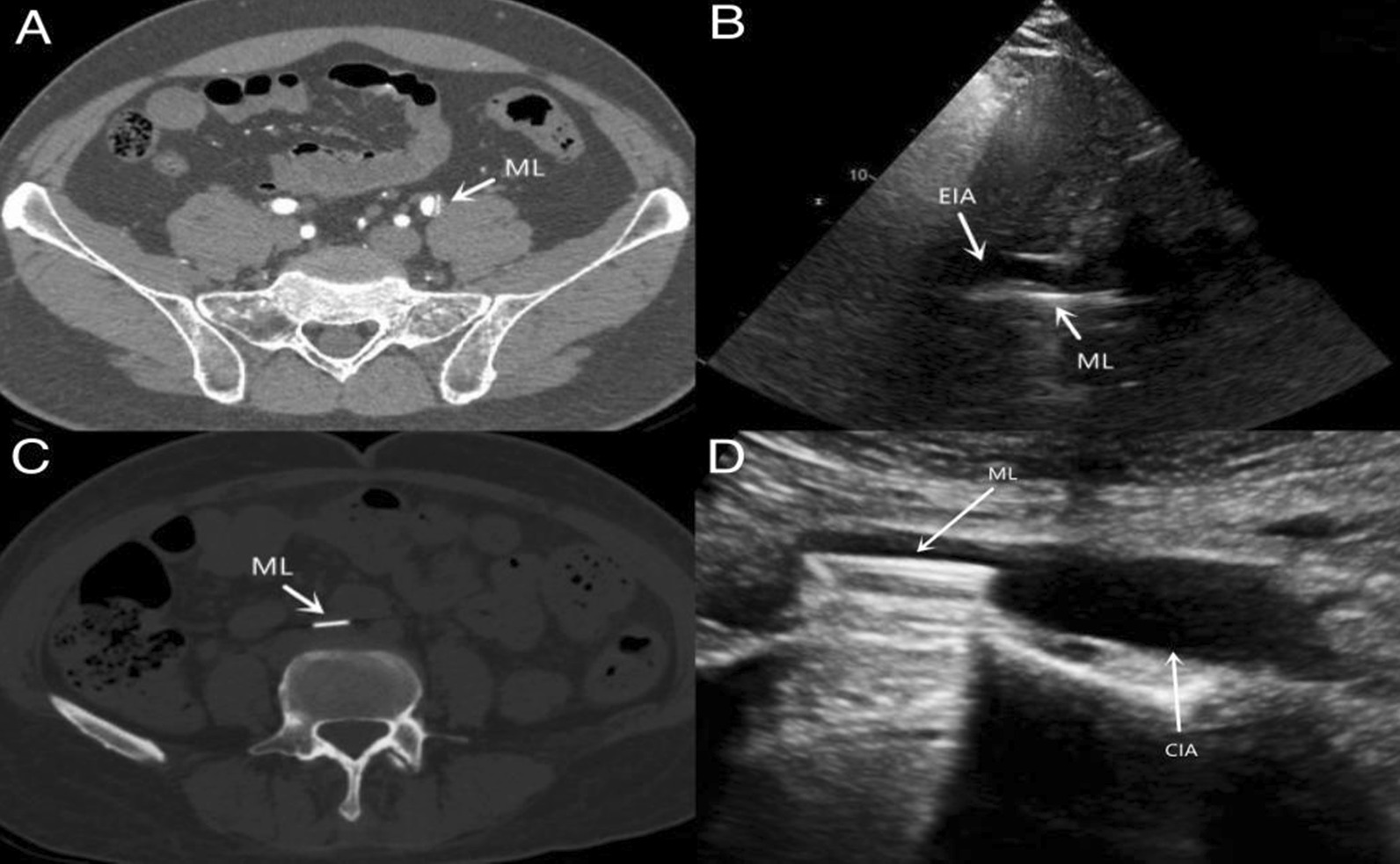
CT and ultrasound data of the escape bileaflet. **A** Ten years after the operation, CT showed that the detached aortic valve leaflet was located in the left external iliac artery, where the blood flow was unobstructed, and there was no obvious stenosis or peripheral hematomas. **B** Ten years after the operation, ultrasound showed that the detached aortic valve leaflet was located in the left external iliac artery, without thrombosis, and there was smooth blood flow; **C** Three months after the operation, CT showed that the detached mitral valve leaflet was located in the right common iliac artery, the leaflet was intact, and no peripheral hematomas were found. **D** Three months after surgery, ultrasound showed that the detached mitral valve leaflet was located in the right common iliac artery, the leaflet was intact, and no thrombosis or peripheral hematoma was found. ML: mechanical flap; EIA: External iliac artery; CIA: Common iliac artery; CT: Computerized tomography

The patient with mechanical aortic valve escape completed the 10-year follow-up, and patient with mechanical mitral valve escape completed the 3-month follow-up. Three months after the operations, color Doppler ultrasounds showed that the average blood flow velocity at the embolic site of the two patients was 122 cm/s, and there was no thrombosis at the embolic site and no obvious hematoma at the embolic site. The ankle brachial index (ABI) of the affected limb was 1.08. Ten years after the operation, a color Doppler ultrasound showed that the blood flow velocity at the embolic site of the aortic valve failure was 125 cm/s; there was no thrombosis or hematoma at the embolic site; the patient had no lower limb symptoms; and the ABI value of the affected limb was 1.07, as shown in Fig. 3.

## Discussion and conclusions

Fuwai Hospital and Guangyuan First People's Hospital have completed 24,710 valve replacements and 3264 s-time valve replacements within a time frame of longer than ten years. Only two patients with unilateral leaflet escape were found, accounting for 0.06% of the cases of second-time valve replacements. This shows that unilateral leaflet escape after mechanical valve replacement is a rare complication. Therefore, we will summarize the two patients in this study, will combine the reports by Nakamura T et al. and will determine the possible causes and treatment strategies for leaflet escape to obtain a reference for scholars and valve companies.

### The clinical manifestations and diagnosis of the patients with unilateral leaflet escape

**Fig. 4:**
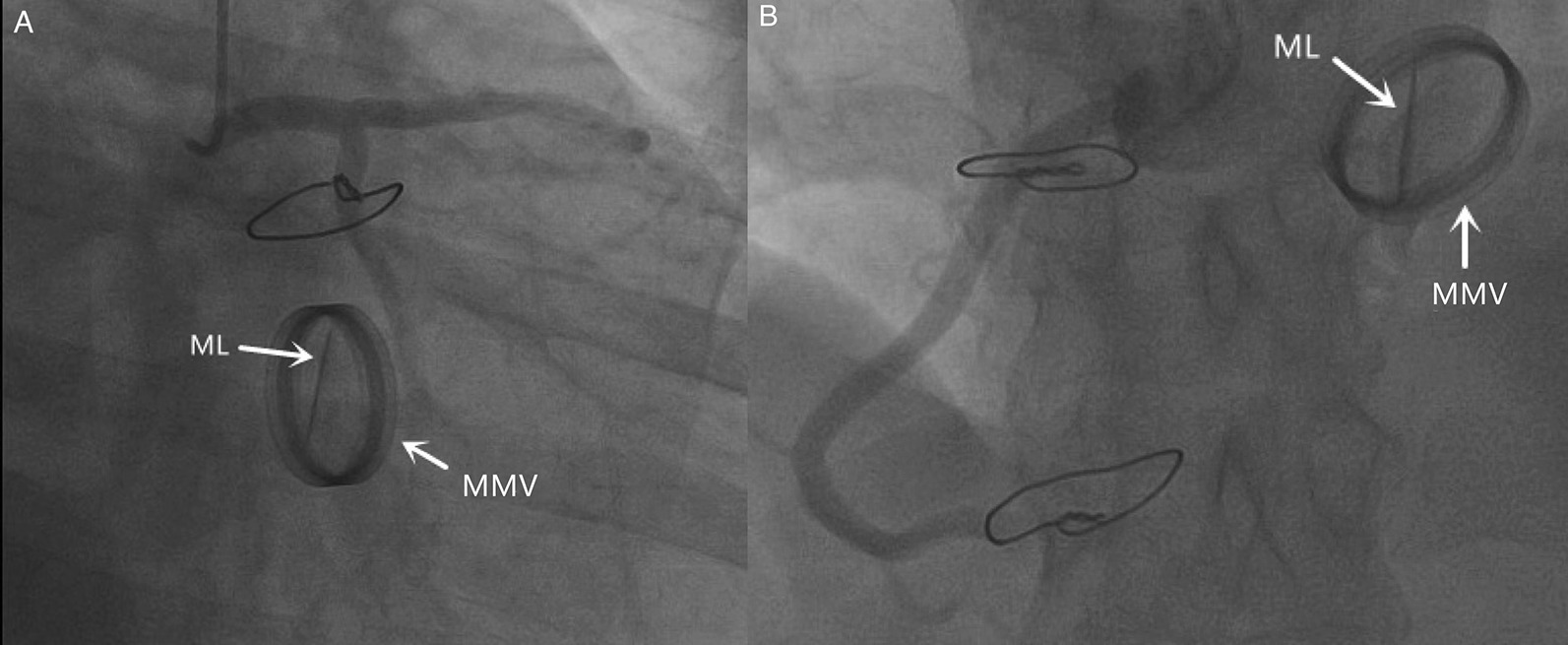
Preoperative coronary angiography of the patient with a mechanical mitral valve. **A** From the perspective of the liver position (RAO 20, CAUDAL 20), we can see the valve opening and closing, and at the same time, we can see that only one leaflet is located on the mechanical valve, but we cannot find the other leaflet. **B** From the perspective of CRANIAL 20, we can see the valve opening and closing, and at the same time, we can see that only one leaflet is located on the valve, but we cannot find the other leaflet. ML: mechanical flap; MMV: mechanical mitral valve; RAO: right front oblique position; CAUDAL: foot position; CRANIAL: head position

Both patients had symptoms, such as recent chest tightness, shortness of breath, and decreased heart function, which suggested that the patients had acute heart failure. The patients’ preoperative ultrasounds had improved, and no valve leaflet escape was detected. Therefore, it is difficult to find leaflet escape and to make a diagnosis by using only cardiac color Doppler ultrasound. However, the patient with aortic valve failure underwent a preoperative CT examination of the entire aorta, and the detached valve leaflet was then found. This also proves that preoperative CT examination of the whole aorta is necessary for such patients. In the report by Nakamura et al., they found that preoperative coronary angiography could clearly show the activity of the valve leaflets, which provided us with options for which to make a diagnosis [7]. From the preoperative angiographic data of the patient with mitral valve disease, we found that from the different angiographic angles, only one valve lobe could clearly be seen (Fig. 4). Therefore, for patients with sudden heart failure who have undergone valve replacement but who have improvements in their cardiac ultrasound, we can further improve the use of whole aorta CT or coronary angiography to facilitate the differential diagnosis of diseases.

### Causes of leaf escape

Susin FM et al.'s in vitro model study showed that when the heart contracts, the forward blood flow rushes out from both sides and from the middle of the valve leaflets, and the side with a smaller opening has a higher forward blood flow velocity after the closing of the valve leaflet [10]. According to this study, a certain degree of turbulence is formed in the sinus, and the pressure in the sinus is greater on the side with small valve opening and closing. This shows that even though the two leaflets are symmetrical in design, the actual opening and closing angles in vivo are still different, which is mainly related to the geometric structure of coronary sinus. This shows that the different forward pressures on the valve leaves lead to different types of losses of the valve leaves. Toshinosuke Akutsu et al. found that the turbulence that is formed in the coronary sinus will counteract the opening and closing of the valve leaflets, which causes pressure on the valve leaflets and reduces their service life [11]. Therefore, the requirements for our industrial design are higher, and it is necessary to adjust the opening and closing angle of the valve leaves by combining the changes in the fluid dynamics and the differences in human anatomy, especially when perfecting the use and design of the valve in animal experiments and in vitro experiments. From the report by Nakamura et al., the valve leaflet leakage that was reported in this report had broken into two pieces that had sharp acute angles, and the patient had lower limb symptoms [7]. However, we found that the leaflets of the two patients in our report were all intact, which indicated that the reasons for unilateral leaflet escape were different. The patient that was reported by Nakamura T could have developed leaflet escape mainly due to material problems of the leaflets, and the leaflets eventually ruptured. However, the two patients in our report may developed leaflet escape due to valvular design problems, which resulted in leaflet escape. The possible reason is the two mechanical valve escape leaflets are intact, and no escape leaflets ruptured, the leaflets material is qualified, and the more likely reason is the valve design failure (such as the angle at which the leaflets open and close) because of insufficient reference to hemodynamic differences. At the same time, we also observed that the fibrous hyperplasia around the valve annulus was serious in two patients, and thrombus was attached in both patients. This will seriously affect the opening and closing of valve leaflets, the effective use area, the pressure of blood flow on valve leaflets and the service life of the valve. This also puts forward higher requirements for valve design, and needs further improvement in anti-thrombosis and anti-tissue proliferation.

### Cardiac management strategy

It is necessary to improve cardiac ultrasound and whole-body CT so that when acute heart failure occurs in a patient, the physicians can determine whether there is valve failure. The research of Nobuyuki Kagiyama et al. and Michael C Scott et al. emphasized the importance of early intravenous drug therapy for acute heart failure [12, 13]. For older patients with high surgical risk scores and who are not good candidates for surgery, diuretics can be used to treat their heart failure. Manantunes and Rajput FA said that patients with mechanical valve failure with a low surgical risk score should undergo a second-time valve replacement as soon as possible [14, 15].

### Treatment strategy of leaflet escape

**Fig. 5:**
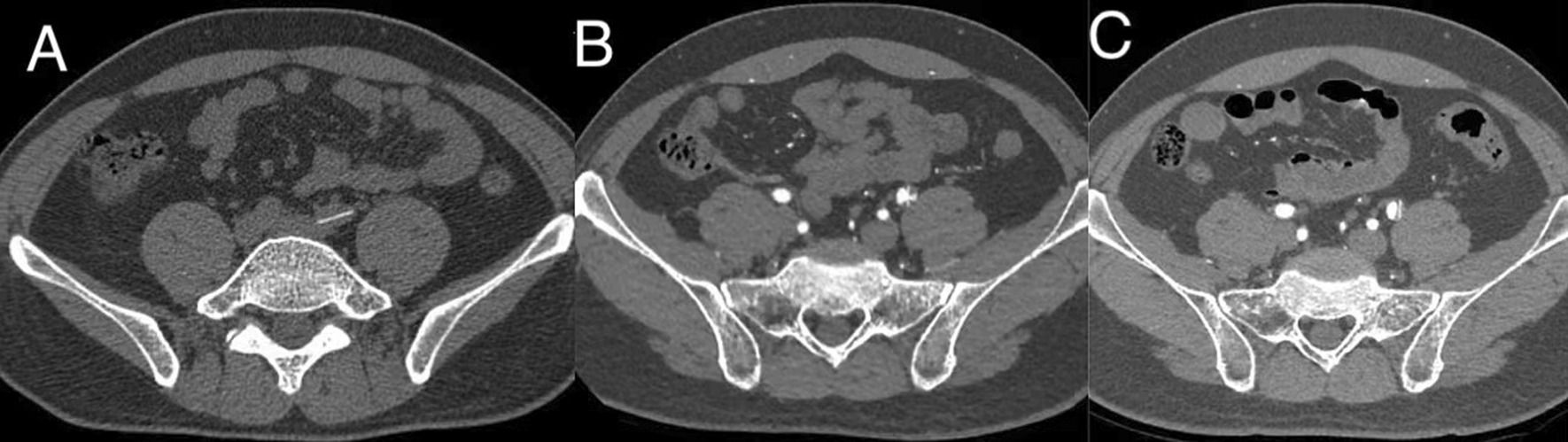
CT images of the aortic valve leaflet displacement over 10 years. **A** In 2010, the valve leaflet was located in the left common iliac artery; **B** In 2016, the valve leaflet was located in the left external iliac artery, and the stenosis at the embolization site was approximately 40%; **C** In 2020, the valve leaflet was embolized in the left external iliac artery, and the stenosis at the embolization site was approximately 30%. CT: Computerized tomography

Mechanical valve leaflet escape is a rare complication, and there is no current treatment for the valve leaflet that has shed. Because the two patients had no any symptoms of lower limbs, they had signed a consent (the patients and their families refused to remove the escaped leaflet). During the 10-year follow-up, we found that the detached leaflets gradually migrated distally. The detached leaflets were initially located in the left common iliac artery, and at the last follow-up, the leaflets were stable in the left external iliac artery, as shown in Fig. 5. Therefore, we conclude that there are three risks. 1. During the 10-year follow-up, thrombosis can develop in patients with embolisms, which may lead to lower limb ischemia. Second, in the process of leaflet escape, there is the possibility of the development of abrasions within blood vessel, leading to blood vessel rupture or acute thrombosis. Third, in the process of the detached leaflets migrating distally, there is the possibility that the valve leaflet can block blood vessels. Nakamura et al. reported cases in which the escape leaflets (leaflet rupture) were surgically removed in time, but the author only reported the cases and did not report on the mid-term and long-term follow-up results. Some published researches in the literature recommend removing the escaped leaflet within the short period of replacing the dysfunctional heart valve in stable conditions [16–19]. Therefore, the advantages and disadvantages of the two treatment schemes can be selected according to a case by case basis, without more evidence-based guidelines. For the patient with mitral valve diseases, we did not perform primary surgical removals of the escape leaflets. The reasons for this are as follows: 1. The patient did not have obvious lower limb symptoms and did not develop any concerning symptoms. 2. Through the examination of CT and color Doppler ultrasound, there were no thrombi or hematomas, the valve leaflets were intact, and the blood flow velocity was not significantly affected. 3. The patients need to take oral warfarin anticoagulation chronically, and this treatment prevents thrombosis; 4. The risk of simultaneous thoracotomy, iliac artery incision and exploration is high, which can increase the perioperative mortality of patients. 5. Carlos Amoros Rivera et al. reported a case of leaflets escape patient not to extract the disc. The patient remains in class I of the NewYork Heart Association classification 6 months after mitral valve replacement and has recovered completely [8]. Therefore, due to the careful monitoring, the patients' symptoms and signs were identified, the findings were combined with the findings of auxiliary examinations, and the treatment plan was adjusted. The escaped leaflet that fractured transversely should be removed as long as the patent in stable conditions.

In summary, unilateral leaflet escape of the bileaflet mechanical prosthetic heart valves after surgery is a rare complication. The reason for valve leaflet escape of the bileaflet mechanical prosthetic heart valves may be related to the leaflet design and leaflet materials of the valves. In the case of acute heart failure after valve replacement, we should be aware of the possibility of valve leaflet escape and the timely use of cardiac color Doppler ultrasound, coronary angiography and whole-body CT before the valve replacement. For the residual in vivo valve leaflets, it is necessary to carefully monitor the valve leaflets for rupture and to evaluate the influence on blood vessels, and ruptured valves that affect the blood flow should be surgically removed. However, patients whose detached valve leaves do not damage the distal blood vessels should be carefully monitored. If there are changes in the detached leaflets, they should be immediately removed by surgery to prevent the occurrence of adverse events.

## Data Availability

For data sharing, please contact the corresponding author of this article.

## Abbreviations

STS: Society of Thoracic Surgeons
NYHA: New York Heart Association
INR: International standard ratio of prothrombin time
BNP: Terminal pro-brain natriuretic peptide
GKS: Beijing Star Medical Device Co., Ltd.
ABI: Ankle-brachial index
MMV: Mechanical mitral valve
PTV: Proliferative tissue of valve
ML: Mechanical flap
EIA: External iliac artery
CIA: Common iliac artery
CT: Computerized tomography
ML: Mechanical flap
MMV: Mechanical mitral valve
RAO: Right front oblique position
CAUDAL: Foot position
CRANIAL: Head position
